# Experiences and perceptions of sexism in dementia research careers: A global cross‐sectional survey

**DOI:** 10.1002/alz.70123

**Published:** 2025-04-12

**Authors:** Adam Smith, Charlèss Dupont, Diana Karamacoska, Sara Laureen Bartels, Elizabeth A. English, Nathan M. D'Cunha, Darina V. Petrovsky, C. Elizabeth Shaaban

**Affiliations:** ^1^ Institute of Neurology University College London London UK; ^2^ Faculty of Medicine and Pharmacy Vrije Universiteit Brussel Ixelles Belgium; ^3^ NICM Health Research Institute and Translational Health Research Institute Western Sydney University Penrith New South Wales Australia; ^4^ Department of Psychiatry and Neuropsychology and Alzheimer Centre Limburg Mental Health & Neuroscience Research Institute Maastricht University Maastricht the Netherlands; ^5^ Yusuf Hamied Department of Chemistry University of Cambridge Cambridge UK; ^6^ UK Dementia Research Institute Cambridge UK; ^7^ School of Rehabilitation and Exercise Sciences Faculty of Health University of Canberra Australian Capital Territory Australia; ^8^ Centre for Ageing Research and Translation Faculty of Health University of Canberra Australian Capital Territory Australia; ^9^ Division of Women Children and Families School of Nursing Duke University Durham North Carolina USA; ^10^ Department of Epidemiology School of Public Health University of Pittsburgh Pittsburgh Pennsylvania USA; ^11^ Alzheimer's Disease Research Center University of Pittsburgh Pittsburgh Pennsylvania USA

**Keywords:** academic careers, Alzheimer's disease, early career researchers, sexism

## Abstract

**INTRODUCTION:**

Sexism is prevalent in academia and is a crucial factor driving women out of the academic workforce. However, sexism in dementia research remains underexplored. This study aimed to understand the perceptions and experiences of early‐career dementia researchers (ECDRs) with sexism in the field.

**METHODS:**

In September/October 2021, a global online survey was conducted targeting ECDRs. The survey assessed their career experiences, including sexism, and was distributed through networks, social media, and e‐mail lists. Responses were analyzed using descriptive and inferential statistics.

**RESULTS:**

Of the 345 respondents, more than half of the female ECDRs (52%) reported facing sexism in their careers, ranging from overt discrimination to subtle biases. Experiences varied by career stage and location, and many ECDRs reported a lack of institutional support.

**DISCUSSION:**

These findings reveal the prevalent nature of sexism in dementia research and highlight the need for targeted interventions to foster a more inclusive research environment.

**Highlights:**

- A global survey revealed 52% of female early‐career dementia researchers experience sexism.
- Subtle sexism, like microaggressions, impacts confidence, and collaboration choices.
- Experiences of sexism vary by geography, with North America reporting higher prevalence.
- Assistant professors report higher rates of institutional and overt sexism.
- Findings emphasize the need for policies addressing implicit bias and sex inequality.

## 1 BACKGROUND

Women comprise a substantial proportion of early‐career researchers, ranging from 30% to 62% depending on university, country, and field. Only 22% to 40% of fixed‐term professorships are held by women, with an even smaller fraction (18%) ascending to university leadership roles.^1^, ^2^, ^3^ Boivin et al. argue that sexism is one of the main reasons for many talented women to leave academia, causing a loss from the research community of scientific expertise and economic potential.^4^

Despite an increasing number of female researchers in early career stages and various initiatives promoting sex equality, sexism—whether overt, covert, or systemic—continues to hinder their progression in academia. A 2021 Danish study involving 5587 respondents on sex‐based violence and harassment in academia found that women undergraduate and PhD students faced elevated levels of sexual harassment and bullying.^5^ Notably, 37% of women who participated reported experiencing at least one instance of verbal sexism, resulting in feelings of exclusion or degradation. In addition, more subtle forms of sexism in academic settings have been reported in several studies,^3^, ^6^, ^7^ including sex biases in hiring practices, disparities in grant allocations, limited publication opportunities, and restricted access to leadership roles.^3^, ^6^, ^7^

Experiences and perceptions of sexism within the workplace affect current professionals in terms of their well‐being and retention within research but may also have implications for attracting new, diverse talent to the field. Regularly monitoring and assessing researchers’ experiences is crucial for identifying successes and areas needing improvement. This ongoing examination enables tracking of progress and pinpointing specific needs for further enhancement.

The career landscape in dementia research, like many fields within science, technology, engineering, and mathematics (STEM), also exhibits sex imbalance, frequently presenting obstacles that disproportionately impact women.^6^, ^7^, ^8^, ^9^, ^10^, ^11^, ^12^ Due to the complex nature of dementia, research in this field is highly interdisciplinary, incorporating biomedical, clinical, social, and public health sciences, among others, meaning that researchers navigate different academic cultures and career pathways. Having a holistic understanding of the state of sexism experiences across the whole dementia research field thus requires investigating sexism across all its subfields. Additionally, early‐career dementia researchers (ECDRs) are at a particularly vulnerable stage in their careers, during which structural barriers and negative experiences may strongly influence their decision to remain in or leave academia. Given that retention issues emerge early, understanding sexism at this stage is key to informing targeted interventions. This study aimed at determining if and how sexism is experienced and perceived specifically in the field of dementia research by ECDRs. The study also evaluated whether there are differences in experiences of men versus women, across career stage, and by geographical location. To answer these questions, survey data were collected and analyzed by University College London (UCL) and the Professional Interest Area to Elevate Early Career Researchers (PEERs) of the Alzheimer's Association International Society to Advance Alzheimer's Research and Treatment (ISTAART).

## 2 METHODS

### 2.1 Study design

This cross‐sectional survey study was conducted online between September and October 2021, using a structured questionnaire in SurveyMonkey. It was aimed at individuals who self‐defined as a current or former (within the last 2 years) ECDR (pre‐tenure). Participants were recruited by promoting the survey on social media, online news items, newsletters, and community engagement via ISTAART. The survey was open worldwide to anyone who self‐defined as an ECDR. The survey asked up to 161 questions related to careers in dementia research, including six questions related to sexism and discrimination.^11^ Participants were permitted to skip questions and were not required to provide names or contact details. The UCL Research Ethics Committee reviewed and approved the study (REC; Number 21275/001). Informed consent was completed by participants on SurveyMonkey before completing the survey. Overarching survey results were published as a policy white paper in April 2022,^11^ and did not include these more detailed analyses we report here.

### 2.2 Survey and data collection

The questionnaire used in this study was developed in consultation with six self‐defined ECDRs from North America, Europe, Australia, Africa, and South America working across various research areas, including basic science, dementia care, public health, and clinical research. The questions and response options were designed to reflect diverse experiences and disciplinary perspectives, ensuring relevance across different fields of dementia research. The survey was then tested with a small sample of 10 additional researchers before being launched. The questionnaire was uniquely designed for this survey of the field, and terminology was informed by best practices from large‐scale surveys, such as those conducted by the UK Office for National Statistics.

The questionnaire was written in English with single‐select or multiple‐select answers. It also included questions to determine social/demographic variables, including sex, location, and research field. Survey logic was built in dictating that some questions were presented to respondents or skipped depending on how they answered prior questions, resulting in a tailored set of questions relevant to the respondent. The questions specifically related to sexism, were (pick‐list reply options defined in results):

1. What gender do you identify as? (man/woman/genderqueer/non‐binary/self‐described—participant completes open‐text field in their own words)—selected to align with UK office of national statistics preferred terms.
2. As an Early Career Researcher, have you ever personally experienced discrimination or prejudice due to: your gender (sexism) in the context of academia/your professional life? (Yes/No).
3. In what way have you experienced sexism? (I would rather not answer/Benevolent sexism/Double standards/Harassment, hostile sexism/Institutional sexism, gender discrimination/Misogyny/Objectification/Online abuse/Religious sexism/Microaggressions/Other). To ensure consistency in interpretation, respondents were provided with brief definitions of different forms of sexism. These aimed to clarify terms such as benevolent sexism (e.g., romanticizing women as objects of heterosexual affection and reinforcing the belief that men must protect women).
4. How has your experience of sexism affected you? (I would rather not answer/I have experienced mental health problems, depression as a result/I have considered leaving my institution/I changed my workplace or institution/It has affected my effectiveness at work/It has impacted my career and delayed my progression/It has affected the way I interact with my coworkers or supervisors/It has affected the way I interact with peers at conferences/It has affected the way I write grant applications and journal submissions/It has affected the way I socialize with peers outside of work/It has affected the way I choose my collaborators/It has affected my confidence/It has affected my motivation/It has affected my ambition/Other).
5. In the context of academia/your professional life, and recognizing that you may or may not have been affected: Do you personally feel that the different forms of discrimination/prejudice (i.e., sexism) are getting better or worse? (Much worse/Worse/Unchanged/Better/Much better/Do not Know, Unable to Answer).

**RESEARCH IN CONTEXT**

1. **Systematic review**: This review examined global literature on sexism in academia, focusing on sex disparities in dementia research careers. No prior study has explored how sexism—overt, covert, or systemic—is experienced by early‐career dementia researchers (ECDRs) globally or how these experiences vary by sex, career stage, or region. This study fills a critical gap, offering insights to inform strategies addressing sexism.
2. **Interpretation**: Over half of female ECDRs (52%) report experiencing sexism, underscoring persistent sex biases in academia. Subtle forms of sexism and perceived institutional unhelpfulness highlight the urgent need for awareness campaigns, implicit bias training, and policy reforms to create inclusive research environments.
3. **Future directions**: Future research should explore cultural and institutional variations, career‐stage differences, and the experiences of gender‐diverse individuals facing sexism and discrimination. Policy‐focused efforts must foster supportive, inclusive environments, enabling all researchers to thrive, and advancing a diverse dementia research workforce.

### 2.3 Data analysis

Descriptive statistics were conducted using SPSS version 29 and SAS version 9.4. First, descriptive analyses were performed to summarize the data, with results presented as numbers and percentages. Bar graphs were used to visualize results, depicting data[Table alz70123-tbl-0001] distributions. Inferential statistical tests were then conducted to examine the relationships and differences within the data. Chi‐square tests were used to analyze categorical variables and determine the significance of observed associations. In SAS, exact chi‐square tests were performed using a Monte Carlo *P* value estimate, 10,000 samples, and a seed set to 15,224. Analyses were conducted to evaluate differences by sex, career stage, and geographical location. Analyses by sex were conducted including only men and women due to low counts of participants of other genders (3% (*n *= 9)). All statistical tests were two‐tailed, and a *P* value of < 0.05 was considered statistically significant.

## 3 RESULTS

### 3.1 Sample characteristics

Of the 584 people who completed the full survey, 345 were included in the analytic dataset as they answered one or more of the questions relating to sexism. In total, 66% (*n* = 229) of these respondents were women; 31% (*n* = 107) men; and 3% (*n* = 9) genderqueer, non‐binary, or self‐identified as another gender. Table 1 displays the relevant sample characteristics of the total survey respondents.

**TABLE 1 alz70123-tbl-0001:** Participant characteristics.

	*n*	%
Age		
< 18–24	33	9.3
25–34	172	49.9
35–44	106	30.7
45–54	23	6.7
55+	10	2.9
Prefer not to answer	1	0.6
Gender		
Woman	229	66.4
Man	107	31.0
Genderqueer/non‐binary/prefer to self‐describe	9	2.6
Location (Country)		
American	62	18.0
Argentinian, Belgian, Danish, French, Mexican, South Korean	4	1.2
Australian	7	2.0
Brazilian	29	8.4
Canadian, Irish	9	2.6
Chinese	15	4.3
Dutch, Nigerian	18	5.2
English	50	14.5
German	14	4.1
Ghanaian, Singaporean, Swedish, Swiss,	2	0.6
Indian	11	3.2
Italian, Portuguese, Spanish,	5	1.4
Scottish	10	2.9
Cameroonian, Chilean, Colombian, Congolese, Costa Rican, Cuban, Cypriot, Czech, Greek, Guyanese, Indonesian, Iranian, Iraqi, Israeli, New Zealander, Norwegian, Peruvian, Polish, Puerto Rican, Rwandan, Salvadorean, Taiwanese, Ugandan, Welsh	1	0.3
Prefer not to answer	15	4.3
Position		
Undergraduate student	18	5.2
PhD/graduate student	122	35.4
Postdoctoral researcher/research fellow	109	31.6
Assistant professor	46	13.3
Associate/ full professor	16	4.7
Other (e.g., lecturer/instructor/MD psychiatrist/research associate/research scientist/trainee)	34	9.9
Research field (multiple selections possible)		
Arts and dementia	21	2.5
Basic sciences and pathogenesis	105	12.7
Biomarkers	116	14.1
Clinical	70	8.5
Communities/environment	32	3.8
Data analysis	89	10.8
Delivery of drug trials	5	0.6
Dementia care	79	9.6
Drug discovery/development	23	2.7
Neuropsychology	73	8.7
Patient and public involvement	36	4.3
Public health	70	8.5
Social care	41	4.9
Technology	33	4.0
Other	29	3.5

### 3.2 Experiencing sexism

The survey asked respondents[Fig alz70123-fig-0001] of[Fig alz70123-fig-0002] all genders “As an Early Career Researcher, have you ever personally experienced discrimination or prejudice due to: your gender (sexism)?” In total, *n* = 340 (*n* = 108 men, *n* = 229 women) survey respondents answered the question and 2% provided a “Rather not answer” reply (*n* = 3 men, *n* = 4 women). Overall, 37% (*n* = 125 people) of respondents reported personally experiencing sexism. For respondents identifying as other genders (*n* = 9), 22% (*n* = 2) responded “Yes,” 22% (*n* = 2) responded “No,” and 55% (*n* = 5) did not respond. While a higher percentage of women (*n* = 120, 52%) reported experiencing sexism, a small number of men (*n* = 5, 5%) also indicated similar experiences. These results differed significantly between men and women (*p* < 0.0001).

**FIGURE 1 alz70123-fig-0001:**
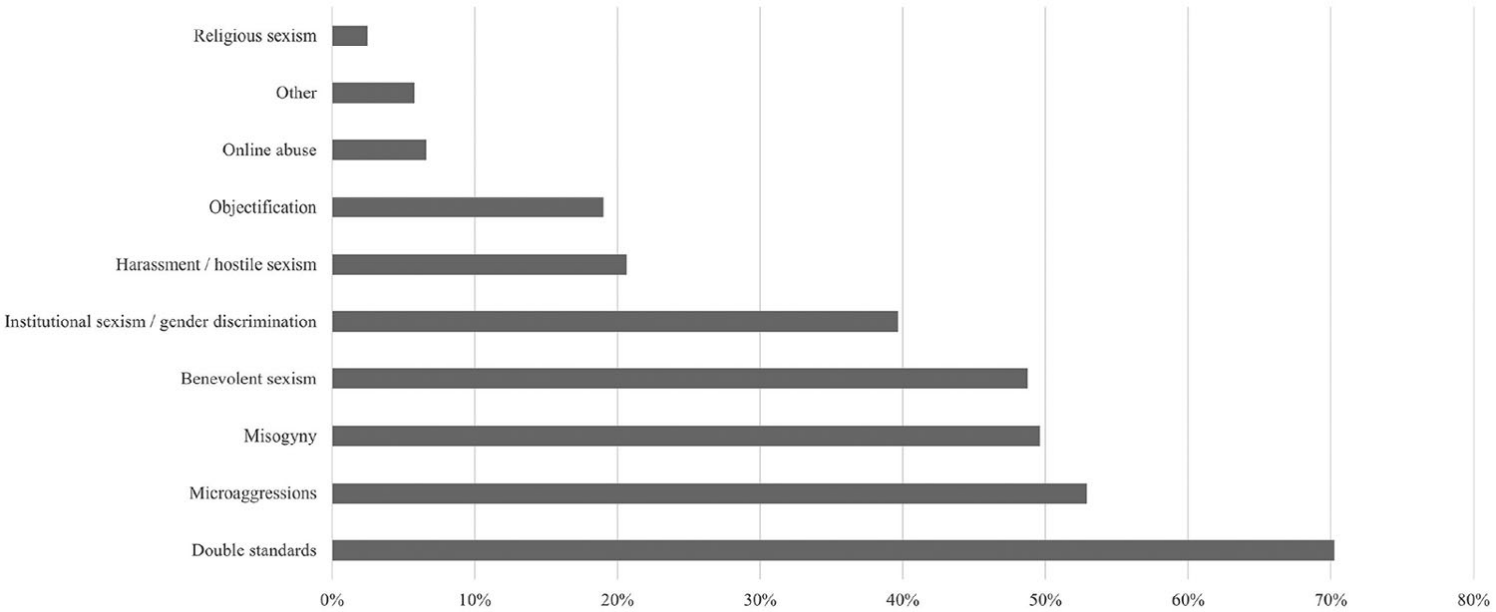
Results of the question “In what way have you experienced sexism?”

All 125 respondents who indicated that they had experienced sexism answered additional questions to help understand their experiences and impacts. The survey asked, “In what way have you experienced sexism?” and respondents were permitted to select as many answers as were relevant from a predefined list (Figure 1). The most experienced form of sexism was “Double standards,” indicated by ≈ 70% of respondents (*n *= 85). “Microaggressions” were the second most common, experienced by 53% of respondents (*n *= 64). “Misogyny” and “Benevolent sexism” (contributing to the stereotype of gender roles) were also prevalent, impacting 50% (*n *= 60) and 49% (*n *= 59) of respondents, respectively. “Institutional sexism/gender discrimination” was experienced by 40% of respondents (*n *= 48). Four out of the five men who experienced sexism reported that they experienced “double standards.”

### 3.3 Experiencing sexism—career stage comparison

**FIGURE 2 alz70123-fig-0002:**
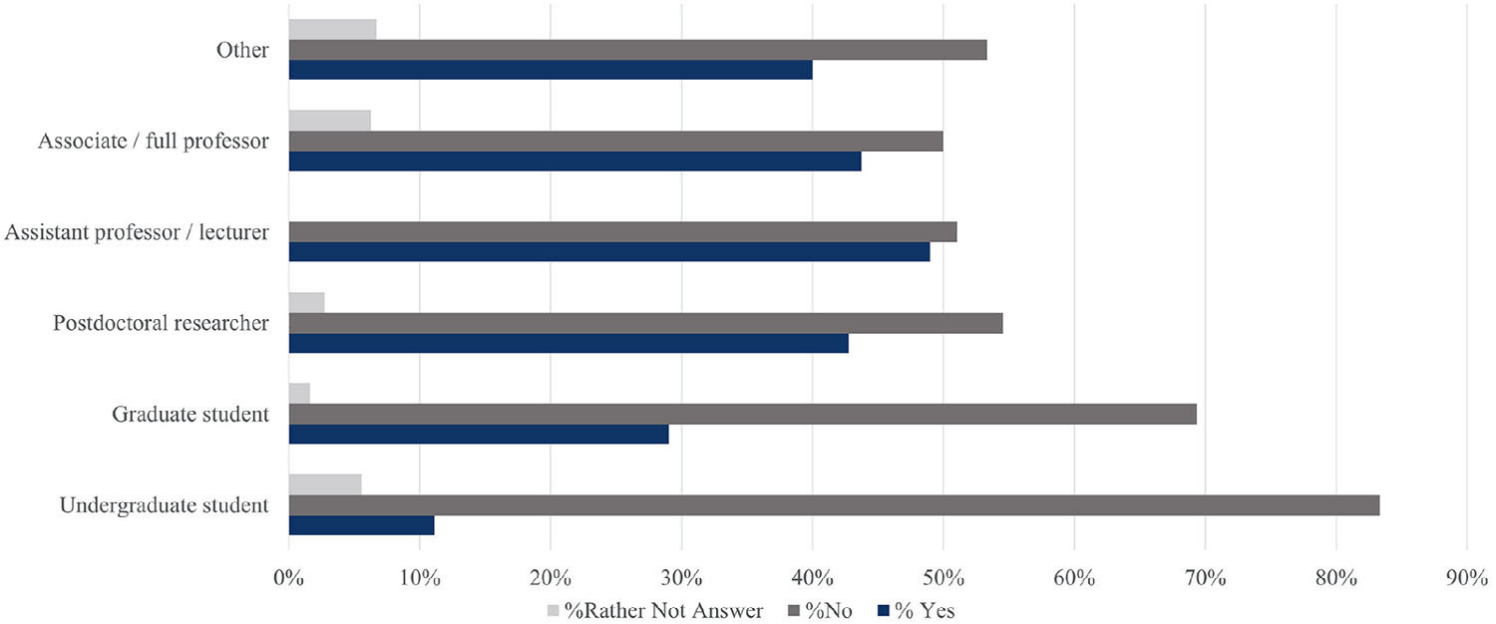
Analysis of early‐career dementia researcher responses to the question “As an Early Career Researcher have you ever personally experienced discrimination or prejudice in your career due to: your gender (sexism)?” by career stage of respondents.

To understand if sexism is universal across all career stages, we analyzed respondents’ experiences (*N *= 345) of sexism by their current career stage (Figure 2). A total of 11% (*n *= 18) of undergraduate students reported experiencing sexism, compared to 43% (*n *= 47) of postdoctoral researchers, and 49% of assistant professors/lecturers (*n *= 25). This difference was statistically significant, with endorsement of sexism experiences overall increasing by career stage (exact chi‐square *p* = 0.047).

Building on the experiences of sexism, we explored how sexism manifested at different career stages (Table 2). A total of 121 respondents answered an additional question relating to how sexism was experienced, with all being permitted to select multiple responses. “Double standards” were not experienced by undergraduates but were experienced by a large majority of graduate students, postdoctoral researchers, and assistant professors (exact chi‐square *p* = 0.001). Microaggressions and misogyny were experienced most commonly by assistant professors and respondents in the “other” career stage (exact chi‐square *p* = 0.004 and *p* = 0.049, respectively). Among respondents experiencing religious sexism, all were graduate students (9% of graduate student's overall experience religious sexism), though this difference by career stage was not statistically significant. Among those experiencing online abuse, most were graduate students (14.3% of graduate students experienced online abuse), but this difference was not significant.

**TABLE 2:** Sexism manifested at different career stages.

	All		Undergraduate students		Graduate students		Postdoctoral researcher		Assistant professor/lecturer		Associate/full professor		Other		
Form of sexism	*N*	%	*N*	%	*N*	%	*N*	%	*N*	%	*N*	%	*N*	%	*p*
Benevolent sexism	59	48.8%	1	50.0%	21	60.0%	21	46.7%	12	57.1%	1	20.0%	3	23.1%	0.17
Double standards	85	70.2%	0	0.0%	28	80.0%	31	68.9%	19	90.5%	2	40.0%	5	38.5%	0.001
Harassment/hostile sexism	25	20.7%	1	50.0%	7	20.0%	6	13.3%	8	38.1%	0	0.0%	3	23.1%	0.17
Institutional sexism/gender discrimination	48	39.7%	1	50.0%	13	37.1%	14	31.1%	12	57.1%	2	40.0%	6	46.2%	0.51
Misogyny	60	49.6%	1	50.0%	16	45.7%	18	40.0%	16	76.2%	1	20.0%	8	61.5%	0.049
Objectification	23	19.0%	0	0.0%	11	31.4%	4	8.9%	4	19.0%	1	20.0%	3	23.1%	0.20
Online abuse	8	6.6%	0	0.0%	5	14.3%	2	4.4%	1	4.8%	0	0.0%	0	0.0%	0.37
Religious sexism	3	2.5%	0	0.0%	3	8.6%	0	0.0%	0	0.0%	0	0.0%	0	0.0%	0.21
Microaggressions	64	52.9%	1	50.0%	18	51.4%	16	35.6%	16	76.2%	2	40.0%	11	84.6%	0.004
Other	7	5.8%	0	0.0%	3	8.6%	3	6.7%	0	0.0%	0	0.0%	1	7.7%	0.76
Total individual respondents	114														

### 3.4 Experiencing sexism—continental comparison

**FIGURE 3 alz70123-fig-0003:**
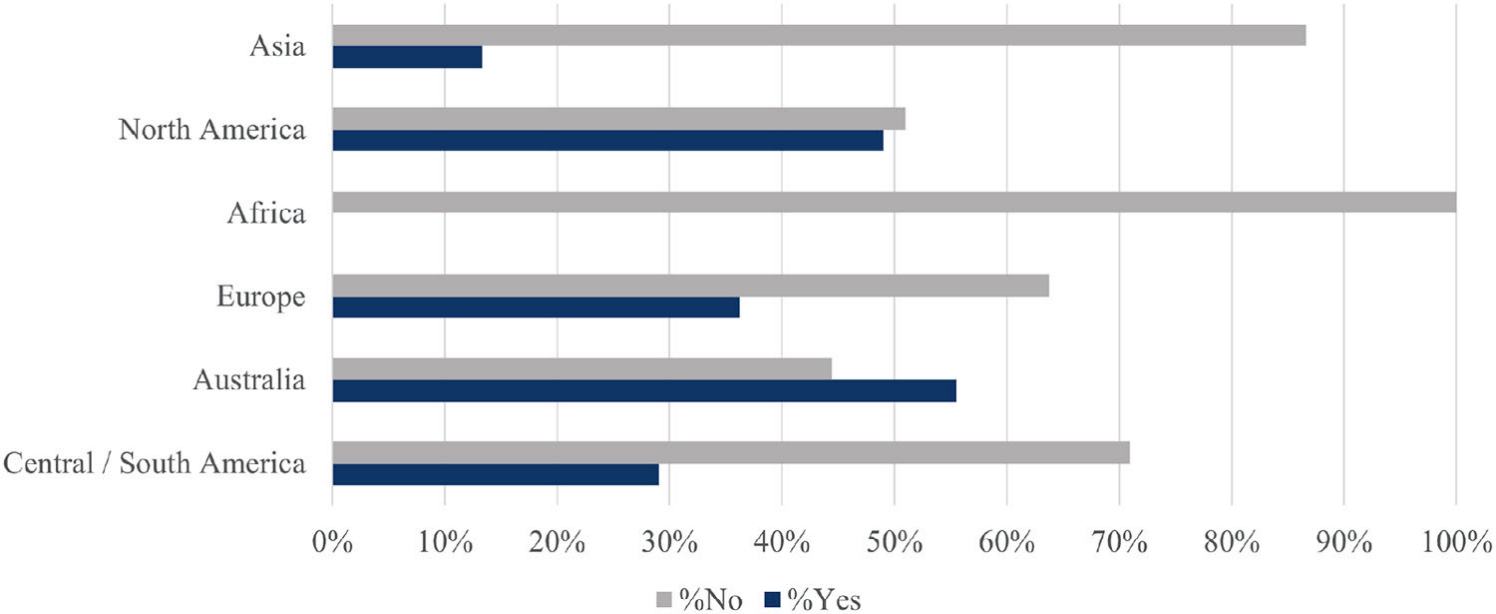
Analysis of early‐career dementia researcher responses to the question “As an Early Career Researcher have you ever personally experienced discrimination or prejudice in your career due to: your gender (sexism)?” by geographical location.

To understand if the issue of sexism exists to the same or lesser degrees in all parts of the world, we further analyzed those reporting experiencing sexism by the continent in which they are currently based (Figure 3). Analysis was only conducted on those who provided a geographic location and answered the specific question related to sexism (*n *= 327). While sexism is influenced by country‐specific laws and workplace policies, a continent‐level comparison was used to capture broader cultural and systemic patterns while maintaining sufficient statistical power for analysis. ECDRs experiencing sexism in the workplace differed by geographic location. There were too few observations from Central/South America (*n *= 31), Africa (*n *= 19), Asia (*n *= 15), and Australia (*n *= 9) to analyze continental differences in more detail. Therefore, only responses from the subgroup of ECDRs working or studying in Europe (*n *= 148) and North America (*n *= 106) were statistically compared. The results suggest that ECDRs in Europe experience sexism less often compared to those based in North America (*p* = 0.046).[Fig alz70123-fig-0003]

### 3.5 Impacts of sexism

**FIGURE 4:**
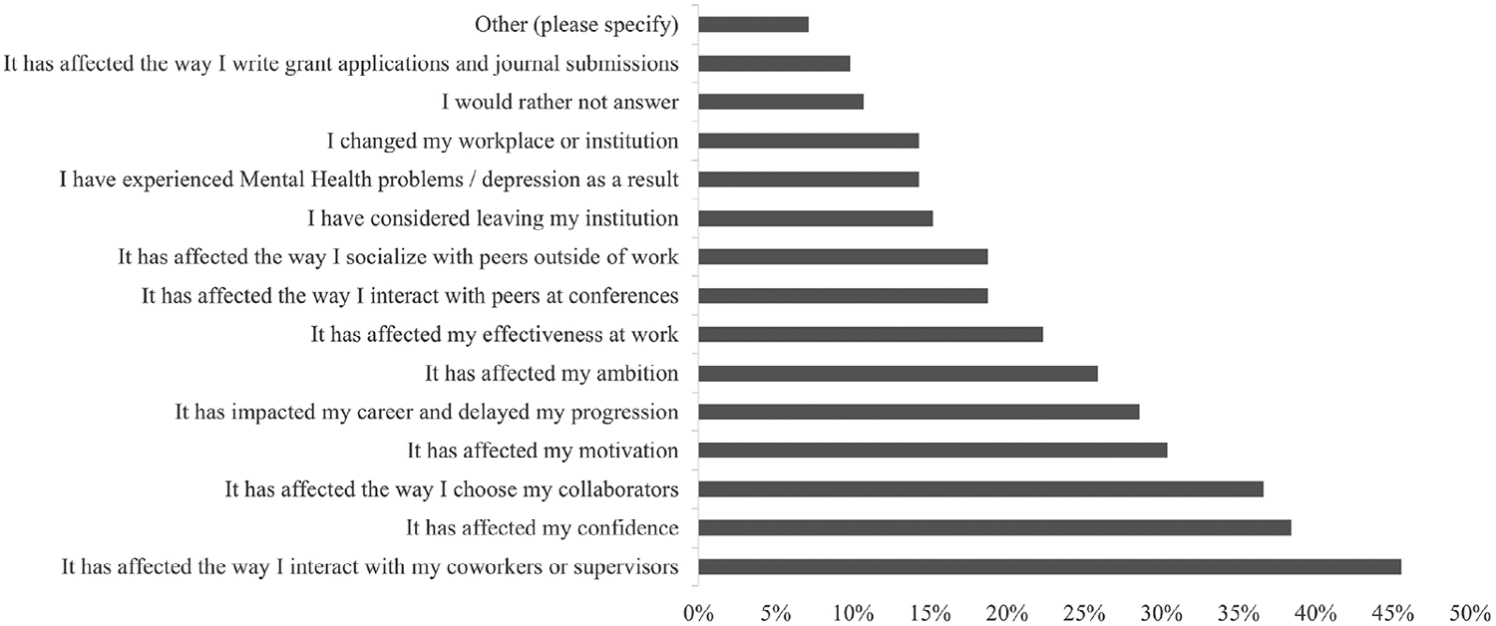
Results of the question “How has your experience of sexism affected you?” by %.

All respondents who indicated had experienced sexism and were invited to answer additional questions about how the experience had affected them, including the question, “How has your experience of sexism affected you?” A total of *n *= 125 respondents answered, and they were permitted to select as many answers as were relevant from a predefined list (Figure 4). The most frequently reported impact of sexism was on “the way individuals interact with their coworkers” experienced by 46% (*n *= 51) of respondents. “Affecting confidence” was experienced by 38% (*n *= 43) of respondents. “Influencing the choice of collaborators” and “affecting motivation” were also notable impacts, reported by ≈ 37% (*n *= 41) and 30% (*n *= 34) of respondents, respectively. “Impact on career and delayed progress” was experienced by 29% (*n *= 32) of respondents. The “Other” impacts of sexism were reported via free‐text entry and included: “Assuming that I as a woman should deal with the pastoral care aspects of supervising students,” “Job interview, hints to potential pregnancy as a problem,” and “I did not let it affect me, and I called out the person responsible.”

A total of *n *= 106 respondents provided data on this question and their career stage (Table 3). For undergraduates, the most acknowledged impacts of sexism were on confidence, motivation, and ambition, each reported by 100% of respondents (*n *= 2). Among postgraduate students, the most common detrimental impact was on confidence, reported by 55% (*n *= 17), followed by changes in interactions with colleagues and supervisors (42%, *n *= 13). Other impacts included effects on collaborator choice, motivation, and ambition (32%, *n *= 10).

**TABLE 3:** Results of the question “How has your experience of sexism affected you?” per career stage.

	Undergraduate students		Graduate students		Postdoctoral researcher		Assistant professor/lecturer		Associate/full professor		Other		
Full answer title	***N***	**%**	***N***	**%**	***N***	**%**	***N***	**%**	***N***	**%**	***N***	**%**	*p*
I would rather not answer	0	0.0%	4	12.9%	5	12.8%	2	10.5%	0	0.0%	1	9.1%	>0.99
I have experienced mental health problems/depression as a result	1	50.0%	6	19.4%	2	5.1%	6	31.6%	0	0.0%	1	9.1%	0.07
I have considered leaving my institution	0	0.0%	4	12.9%	4	10.3%	7	36.8%	1	25.0%	1	9.1%	0.13
I changed my workplace or institution	0	0.0%	1	3.2%	3	7.7%	6	31.6%	2	50.0%	4	36.4%	0.007
It has affected my effectiveness at work	0	0.0%	5	16.1%	8	20.5%	6	31.6%	2	50.0%	4	36.4%	0.40
It has impacted my career and delayed my progression	0	0.0%	7	22.6%	10	25.6%	10	52.6%	1	25.0%	4	36.4%	0.23
It has affected the way I interact with my coworkers or supervisors	1	50.0%	13	41.9%	16	41.0%	12	63.2%	2	50.0%	7	63.6%	0.58
It has affected the way I interact with peers at conferences	1	50.0%	7	22.6%	5	12.8%	7	36.8%	0	0.0%	1	9.1%	0.16
It has affected the way I write grant applications and journal submissions	0	0.0%	2	6.5%	4	10.3%	5	26.3%	0	0.0%	0	0.0%	0.17
It has affected the way I socialize with peers outside of work	0	0.0%	5	16.1%	7	17.9%	7	36.8%	0	0.0%	2	18.2%	0.36
It has affected the way I choose my collaborators	0	0.0%	10	32.3%	11	28.2%	11	57.9%	1	25.0%	8	72.7%	0.02
It has affected my confidence	2	100.0%	17	54.8%	9	23.1%	9	47.4%	2	50.0%	4	36.4%	0.04
It has affected my motivation	2	100.0%	10	32.3%	9	23.1%	6	31.6%	3	75.0%	4	36.4%	0.10
It has affected my ambition	2	100.0%	10	32.3%	9	23.1%	4	21.1%	1	25.0%	3	27.3%	0.28
Other (please specify)	0	0.0%	4	12.9%	2	5.1%	1	5.3%	0	0.0%	1	9.1%	0.80
Total individual respondents	113												

Among postdoctoral researchers, the most commonly reported impacts were changes in interactions with colleagues and supervisors (41%, *n* = 16), followed by influences on collaborator choice (28%, *n* = 11); delays in career progression (26%, *n* = 10); and reductions in confidence, motivation, and ambition (each 23%, *n* = 9). Assistant professors reported a broader range of impacts, with the most common being changes in interactions with coworkers and supervisors (63%, *n *= 12), followed by effects on collaborator choice (58%, *n *= 11) and career progression (53%, *n *= 10). Ambition was less commonly affected compared to earlier career stages. For associate professors, the most frequently reported impact was on motivation (75%, *n *= 3), with confidence, colleague interactions, and work effectiveness each reported by 50% (*n *= 2). Half of associate professors (*n *= 2) indicated they had left their workplace due to sexism, while 25% (*n *= 1) considered leaving. Impacts of sexism that differed significantly by career stage included changing institutions, most commonly reported by associate professors (exact chi‐square *p* = 0.007); choice of collaborators, most commonly reported by assistant professors and those in the “other” career stage (exact chi‐square *p* = 0.02); and confidence, most commonly reported by undergraduate and postgraduate students (exact chi‐square *p* = 0.04).

### 3.6 Acting on sexism

The respondents who indicated that they had experienced sexism (*n *= 125) were asked, “If you raised this with your employer/institution, how helpful were they in addressing it?” A total of 94% (*n *= 118) provided a response (Table 4). Despite the anonymity of the survey, 56% (*n *= 66) selected, “I would rather not answer/not applicable,” highlighting a significant reluctance to discuss the issue. Among those who provided a definitive response, a 5‐fold disparity was observed between those who found their institution helpful versus unhelpful. Only 3% (*n *= 4) rated their employer or institution as “extremely helpful,” and 3% (*n *= 3) as “very helpful,” compared to 16% (*n *= 16) who rated them as “not at all helpful,”’ and 15% (*n *= 18) as “not so helpful.”

**TABLE 4:** Results of the question “If you raised this with your employer/institution, how helpful were they in addressing it?”

If you raised this with your employer/institution, how helpful were they in addressing it?	Responses	
	*N*	%
Extremely helpful	4	3.39
Very helpful	3	2.54
Somewhat helpful	8	6.78
Not so helpful	18	15.25
Not at all helpful	19	16.1
I would rather not answer/not applicable	66	118

### 3.7 Men's versus women's perspectives of changes in sexism

The survey asked respondents “Recognizing that you may or may not have been affected, do you personally feel that the issues mentioned above [i.e., sexism] are getting better or worse, in the context of academia/your professional life?” In total, *n* = 332 (*n* = 105 men, *n* = 227 women) answered the question, and differences in perceptions of changes in sexism between men and women were significant (exact chi = square *p* < 0.0001). Women were likelier than men to feel that things were “much worse” (*n* = 3 [1.3%] vs. *n* = 1 [1.0%]), “worse” (*n* = 20 [9%] vs. *n* = 5 [5%]), “unchanged” (*n* = 81 [36%] vs. *n* = 26 [25%]), or “better” (*n* = 86 [38%] vs. *n* = 26 [25%]), while they less likely than men to report that things were “much better” (*n* = 11 [5%] vs. *n* = 10 [10%]) or to respond as “do not know/unable to answer” (*n* = 26 [11%] vs. *n* = 37 [35%]).

## 4 DISCUSSION

This study explored how ECDRs perceive and experience sexism in dementia research workplaces. In the present sample (*N *= 345), more than one third (37%) of respondents overall and more than one half (52%) of surveyed female ECDRs reported experiencing sexism in their careers. The most common forms of sexism reported were double standards (70%) and microaggressions (53%). The greatest impacts of sexism included negative effects on interactions with coworkers (46%) and reduced confidence (38%). Understanding how ECDRs perceive and experience sexism is crucial: failure to address sexism can lead to the collapse of critical collaborations and the loss of talented researchers^13^ observed with continued sex gaps toward senior career stages. Taken together, this likely impedes dementia research progress.

As sexism is a known problem in academia, recent actions have been taken to fight sex/gender‐based discrimination. An informal review of 50 university websites from across the world found that all universities had specific policies relating to sexism.^14^ Despite these increasing efforts to promote sex equality and inclusivity in academia and research fields,^15^ the persistence of sexism remains alarming in the field of dementia research. The lack of impact of current policies is also shown in our study, as a total of 31% of participants felt their employer or institution was “not at all helpful” or “not so helpful” and only 6% felt their employer or institution was helpful when reporting incidents of sexism. Noticeably, most of the surveyed ECDRs (56%) responded with “I would rather not answer/not applicable” when asked if their institution was helpful, potentially indicating that issues are often not raised internally. This reluctance may stem from factors commonly reported in the wider literature, including shame, fear of repercussions, and mistrust in institutional processes.^16^ Additionally, perceived insignificance—either because individuals themselves downplay the experience or because they anticipate that their institution will not take it seriously—may contribute to under‐reporting.

Our results suggest that men and women differ in their perceptions of whether sexism in dementia research is improving, with men more likely than women to indicate that things have gotten much better or to state that they do not know. Although this difference was statistically significant, the small number of responses at the extremes of the scale limits strong conclusions. This finding suggests a possible disconnect in how men and women perceive progress on sexism, which may have implications for workplace culture and institutional policies. Understanding differences in how men and women perceive sexism in academia is particularly important, given the role of senior leadership in shaping policies, funding, and institutional priorities, and the disproportionate representation of men in leadership position. If men in leadership roles believe sexism has been largely resolved, there is a risk that ongoing issues may not receive adequate attention. Future work with larger sample sizes should confirm these results. Acknowledging these differing perspectives can help foster more equitable and respectful research environments.

The results show that ECDRs have experienced a wide range of types of sexism, from more overt forms like harassment and misogyny to more subtle forms like benevolent sexism and microaggressions. This variety indicates that sexism is a multifaceted issue, affecting individuals in diverse and complex ways. While respondents were provided with brief definitions of the different types of sexism assessed in the survey, individual interpretations may still have varied. This should be considered when interpreting the findings, as personal experiences and contextual factors may shape how respondents identified and categorized instances of sexism. Notably, less commonly discussed forms,^17^ such as microaggressions and benevolent sexism, were reported by ≈ 50% of respondents. While more overt forms like objectification and outward harassment were also present in our study, they were less prevalent. This observation may suggest that institutional policies may have had a greater impact on addressing the more easily identifiable forms of sexism, as discussed in previous literature.^18^ However, the results underscore the significance of recognizing and addressing subtler forms of sexism, including implicit bias. These forms, often overlooked, can perpetuate sex inequality and hinder progress toward creating truly inclusive environments.^15^, ^18^, ^19^ Beyond identifying the forms of sexism most prevalent at each career stage, these findings suggest that institutions should develop targeted interventions. This could include leadership training on implicit bias, mentorship schemes for women and minoritized researchers, and clearer reporting mechanisms to improve institutional responses. Additionally, fostering a culture in which speaking out against sexism is encouraged and supported may help mitigate under‐reporting and improve institutional accountability. By acknowledging and actively addressing these subtler manifestations of sexism, institutions can create more effective strategies for promoting sex equality and fostering a supportive and respectful environment.

Career stage–stratified analysis of sexism is crucial to understand the contribution of sexism to the disproportionate loss of women toward senior career stages. Several forms of sexism, such as double standards, microaggressions, and misogyny, were statistically significantly more commonly experienced at more advanced career stages. On the other hand, although differences in online sexist abuse and religious sexism by career stage were not statistically significant, we found that graduate students were likelier to experience these types of sexism than ECDRs at more advanced career stages. This suggests that these forms of sexism may be increasing in the current climate and should be topics of further research and monitoring.

The present results also show that there is a geographical variation in the experiences of sexism among dementia researchers. This finding suggests that cultural, societal, and institutional factors in different regions may play a role in the expression and notion of sexism. North America (49%) and Australia (56%) stand out for their relatively high percentages of researchers reporting sexism (although we point out that the sample size from Australia was small), compared to Europe (36%). Further exploration is needed to understand the underlying factors contributing to these high rates in specific locations and gain further insights into other world areas (e.g., Africa, Central and South America, Asia).

### 4.1 Limitations and strengths

This cross‐sectional study examined the impact of sexism on ECDRs, shedding light on the prevalence and multifaceted nature of their experiences and the ensuing repercussions.

Several limitations should be kept in mind when interpreting these results. First, the small sample size overall, and particularly for those of other genders, precluded their perspectives and experiences from being adequately represented in our analysis. Future research should prioritize engaging with ECDRs from these gender identities in large enough sample sizes to ensure their voices are heard, and their experiences are meaningfully accounted for. Second, our survey did not inquire about the timing or duration of experiences of sexism, making it difficult to discern at which career stage these incidents occurred. This limitation highlights the need for further research to explore the temporal dynamics of sexism in more detail. Third, there is error in the measurement of sexism; this study examines perceptions of sexism rather than the actual incidence of sexism. Some forms of sexism may be so normalized that respondents may not always recognize them as such, meaning that self‐reported experiences may not capture the full extent of the issue. The ability to recognize and report sexism may also depend on factors such as age, career stage, or prior exposure to discussions on sex bias, which could partially explain some of the subgroup differences observed. Fourth, variations in sample sizes for different survey questions, along with less informative response options such as “do not know” or “I would rather not answer,” may have influenced the comprehensiveness and accuracy of our findings. Fifth, ECDRs self‐defined as being early in their career in responding to the survey call and no cut‐off was used when processing data, leading to a small number of professors and other positions potentially considered more senior being included in the sample. Prospectively, to understand sexism in the field of dementia, a larger sample encompassing more balanced sample sizes across each career stage could be conducted, also controlling for years working in the field. Future studies may expand on these findings by using longitudinal designs to track changes over time or advanced modeling techniques to identify key predictors, mediators, and moderators of sexism. Finally, it is important to point out that examining differences in sexism experiences by continent glosses over heterogeneity at smaller geographical units. For example, national policies, cultural differences, and so forth, likely shape experiences differently within continents.

This study underscores persistent sex biases within the field of dementia research and highlights the need for more effective strategies to combat sexism and improve the retention of women in this field. The differences in perceptions between men and women, along with a great variety of subtler forms of sexism found in this study, emphasize the importance of raising awareness, and addressing implicit bias. Further exploration into variations in experiences of sexism by career stage and geographical location is warranted to understand underlying factors. Future studies should include larger sample sizes across diverse participant characteristics such as race, ethnicity, age, and the like to allow the field to better understand whether certain subgroups of ECDRs are differentially impacted by sexism.

## CONFLICT OF INTEREST STATEMENT

The authors of this manuscript were facilitated by the Alzheimer's Association International Society to Advance Alzheimer's Research and Treatment through the Professional Interest Area to Elevate Early Career Researchers. CES reports that she is the Co‐Chair of the ISTAART Sex and Gender Interest Group, Diversity and Disparities Professional Interest Area, and a member of the ISTAART Advisory Council. Author disclosures are available in the supporting information.

## CONSENT STATEMENT

The survey was approved by the University College London's (UCL) Ethics Review Board (approval number: 21275/001). The requirement for written consent was waived by UCL due to the nature of the survey form. Respondents had to read the survey information before starting the survey and tick a box to confirm that they met the eligibility criteria, and this constituted agreement to participate in the survey.

## Supporting information



Supporting Information
